# Accuracy of One-Piece vs. Segmented Three-Dimensional Printed Transfer Trays for Indirect Bracket Placement

**DOI:** 10.3390/dj12110352

**Published:** 2024-10-31

**Authors:** Bayan Alyammahi, Amar Hassan Khamis, Ahmed Ghoneima

**Affiliations:** 1 Department of Orthodontics and Paediatric Dentistry, Hamdan Bin Mohammed College of Dental Medicine (HBMCDM), Mohammed Bin Rashid University of Medicine and Health Sciences, Dubai P.O. Box 505055, United Arab Emirates; bayan.alyammahi@dubaihealth.ae (B.A.);; 2 Department of Orthodontics and Oral Facial Genetics, Indiana University School of Dentistry, Indianapolis, IN 46202, USA

**Keywords:** indirect bonding techniques, transfer trays, bracket placement, 3D-printing, dentistry, orthodontics

## Abstract

Objective: To assess the accuracy of three-dimensional (3D) printed one-piece vs. multiple segmented transfer trays for indirect bonding techniques in moderate and severe crowding cases. Methods: Eighty digital maxillary dental models were produced by an extraoral scanner. 3D-printed one-piece and segmented trays were virtually designed utilizing Maestro 3D Ortho Studio^®^ v4 and printed using a NextDent printer. The sample was classified into two groups: Group 1 (moderate crowding) included 40 digital models with a space deficiency of 6–7 mm, and Group 2 (severe crowding) included 40 digital models with a space deficiency of 10 mm. Ortho classic brackets were then placed into the 3D printed models with the aid of the transfer trays, and the models with the final bracket positioning were scanned using iTero scanner. Four measurements were selected on each tooth to perform the analysis. Mann–Whitney and Kruskal–Wallis tests were used for comparisons. A *p*-value of ≤ 0.05 was considered statistically significant. Results: In the moderate crowding group, statistically significant differences were detected between the one-piece, segmented, and control groups for three measurements (*p* < 0.001), while the rest of the measurements showed no significant differences (*p* > 0.05). In the severe crowding group, no significant differences were detected for any of the measurements. Conclusions: One-piece and segmented 3D-printed transfer trays are considered accurate tools for indirect bonding in moderate and severe malocclusion cases. The severity of crowding did not affect the accuracy of bracket transfer in indirect bonding.

## 1. Introduction

Accurate placement of orthodontic brackets is a crucial step in orthodontic treatment. Correct alignment of crowns and roots and level marginal ridges are hallmarks of successful orthodontic case management. Inaccurate bracket placement results in incorrect tooth positioning and longer treatment time with a less-than-ideal final occlusion. Positioning orthodontic brackets on tooth surfaces can be performed directly or indirectly [1]. It is considered a technically sensitive procedure that requires special attention to details such as the bonding technique, quality of the bonding materials, and obtaining a dry working field. The fact that a tooth surface may not be accessible because of severe crowding might also complicate the process of direct bonding technique [2,3].

In an attempt to avoid the drawbacks of the direct bonding technique, Silverman and Cohen proposed indirect bonding (IDB) for the first time in 1972 [4,5]. The IDB procedure consists of two stages. Brackets are attached to the patient’s study models in the laboratory stage and then fitted to the tooth surface with the use of a customized tray in the clinical stage. Both bracket failure and clinical performance are not significantly different from conventional bonding technique [6,7]. In the traditional IDB technique, brackets are manually fitted on stone or resin models, and then a transfer tray made from either silicone-based material, vacuum-formed thermoplastic sheets, or a combination of both can be used. A silicone-based tray offers better precision than a thermoform tray [8,9,10,11].

Several digital indirect bonding systems have recently emerged as an outcome of the recent advances in three-dimensional (3D) imaging technology and the development of new software programs. Using these new software programs enables clinicians to simulate tooth movement, align the teeth in their ideal position, virtually position the brackets, and design and print customized transfer trays for the IDB. These software programs involve a built-in bracket library that includes a variety of bracket brands, sizes, and designs where the clinician can choose the right set of brackets to be used. The software programs also provide visualizing tools that allow clinicians to rotate and manipulate the digital models in 360 degrees, previewing the tooth from multiple perspectives to ensure the most accurate bracket placement. The digitally positioned brackets can then be transferred using different approaches. The first approach involves printing the virtual set up including the digital model with brackets that were virtually placed on the teeth. Then, the bracket transfer trays are fabricated in the lab using silicone or vacuum-formed materials. The second approach involves designing the transfer trays or a single jig virtually. Then, these virtual trays can be printed using a biocompatible resin, without the need for an intermediary physical bracket transfer model. In either approach, once the transfer trays have been fabricated, they can be filled with the physical brackets, which are then transferred to the patient’s teeth to be used for IDB [12,13,14,15]. The aim of the current study was to assess the accuracy of using 3D-printed one-piece vs. segmented transfer trays for IDB in moderate and severe crowding cases. We hypothesized that there would be no significant difference between one-piece and segmented 3D-printed transfer trays for indirect bracket placement.

## 2. Materials and Methods

**Figure 1:**
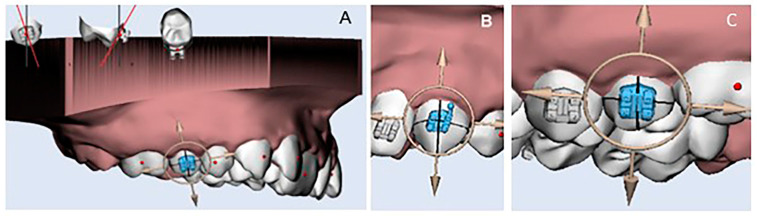
Virtual brackets placement (**A**) on the upper right second premolar, (**B**) upper left canine, and (**C**) upper left second premolar using Maestro 3D Ortho Studio^®^.

The sample consisted of 80 upper dental digital models classified into two groups according to the amount of space deficiency. Group 1 (moderate crowding) included 40 models with a space deficiency of 6–7 mm, and Group 2 (severe crowding) included 40 models with a space deficiency of 10 mm. Dental models of poor quality, primary or mixed dentition, broken teeth, defects or restorations that involved the labial/buccal tooth surfaces were excluded. Models with severe rotation, overlapping anterior teeth, or partially erupted teeth were included in Group 2. The digital models were created by scanning the stone models using the Ortho-Insight 3D Desktop Scanner (Motion View Software LLC, Chattanooga, TN, USA) then saved in stereolithography (STL) format. The STL models were then transported to Maestro 3D Ortho Studio^®^ (AGE Solutions^®^, Pontederia, Italy) software for virtual brackets placement (Figure 1). The ortho Classic Brackets (Roth 0.22′) used in this study were selected from the virtual bracket library of the Maestro 3D Ortho Studio software^®^ and digitally placed on the central incisors, laterals, canines, and premolars of the 3D models. Proper bracket positioning with fine adjustments were verified in the three planes of space. The height, angulation, and mesiodistal tip of each bracket were standardized. All procedures were performed by the same investigator. Digital models after bracket placement were considered the control for comparing the accuracy of bracket positioning.

**Figure 2:**
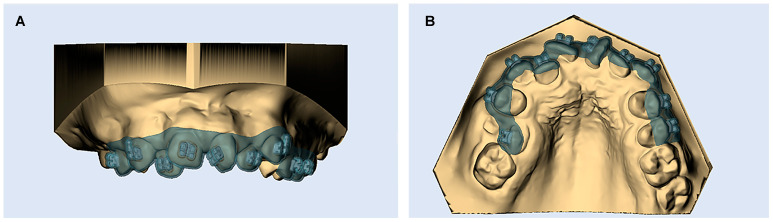
(**A**) Virtual bracket transfer tray. (**B**) Trays designed to cover the buccal aspects of each tooth. The lingual aspects of the teeth were not covered.

**Figure 3:**
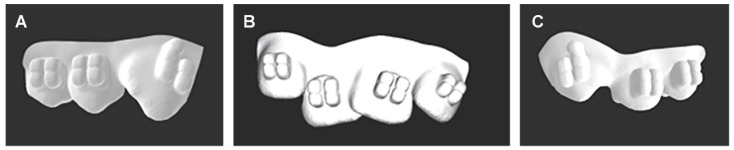
Additional cuts were made virtually between the lateral incisors and canines on both sides using the 3D-Sprint software (3D systems, Rock Hill, SC, USA) for creating three-piece transfer trays, (**A**) for the upper right segment from canine to second premolar, (**B**) for central and lateral incisors, and (**C**) for the upper left segment from canine to second premolar.

The Maestro 3D Ortho Studio^®^ software v4 was used for designing the digital transfer trays with a thickness of 0.5 mm. All trays were designed to cover the buccal cusps of the posterior teeth and incisal edges of the anterior teeth then extend vertically just below the gingival margins from the labial and buccal aspects of each tooth. The lingual aspects of posterior and anterior teeth were not covered (Figure 2). For the segmented transfer trays, additional cuts at the contact areas between the lateral incisors and canines were virtually made on both sides using the 3D printer software (3D-Sprint, 3D systems, Rock Hill, SC, USA) for creating three-piece transfer trays (Figure 3).

All STL files for the digital models and brackets transfer trays were printed with the 3D printer NeXTDent 5100 (NextDent B.V., Soesterberg, The Netherlands) using the light-cured resin material (NextDent Model 2.0, NextDent B.V., Soesterberg, The Netherlands) for the models and (NextDent Ortho Flex, NextDent B.V., Soesterberg, The Netherlands) for the transfer trays. The printing time was 35 min. All models and transfer trays were virtually positioned perpendicular to the platform of the 3D printer. The resin models were subsequently thoroughly cleaned from adhesive resin material using 99% alcohol rinse. LC-3DPrint Box (NextDent B.V., Soesterberg, The Netherlands) was adjusted at 30 min at 60 °C for NextDent Ortho Flex resin, and 10 min at 60 °C for the NextDent Model 2.0. The same process was repeated for all printed trays to ensure consistency and standardization. Brackets were manually inserted inside the transfer trays and each tray was then fitted over the printed model to inspect for proper fitting.

For IDB, a thin layer of Adler Single Bond 2 adhesive (3M Unitec, Monrovia, CA, USA) was applied to the buccal surface and lightly cured. Then, a small amount of Trans bond XT light-curing bracket adhesive (3M Unitec, Monrovia, CA, USA) was applied to the base of the brackets. The trays were carefully positioned over the printed resin models and seated using light finger pressure on the occlusal surface. The adhesive resin was then light cured for 30 s per tooth in all directions. With the use of a dental probe instrument, the trays were carefully removed from the printed resin models.

**Figure 4:**
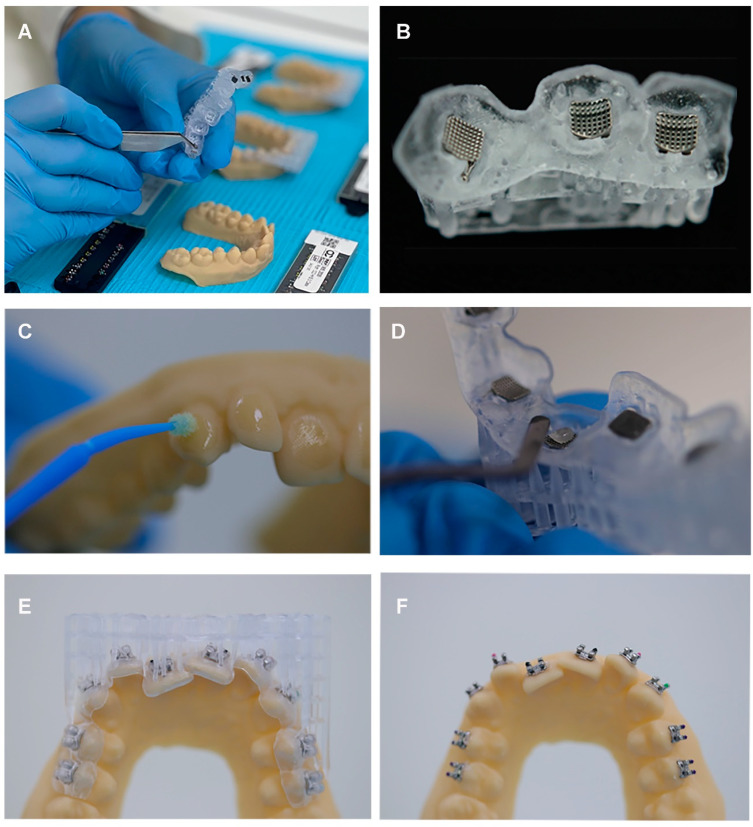
(**A**) Brackets were manually inserted inside the transfer trays, (**B**) loaded trays were checked to ensure proper bracket fitting inside the transfer trays, (**C**) a thin layer of adhesive was applied to the buccal surface, (**D**) bracket adhesive was applied to the base of the brackets, (**E**) trays were carefully positioned over the 3D printed resin models, (**F**) Final bracket positions on the resin model after curing.

**Figure 5:**
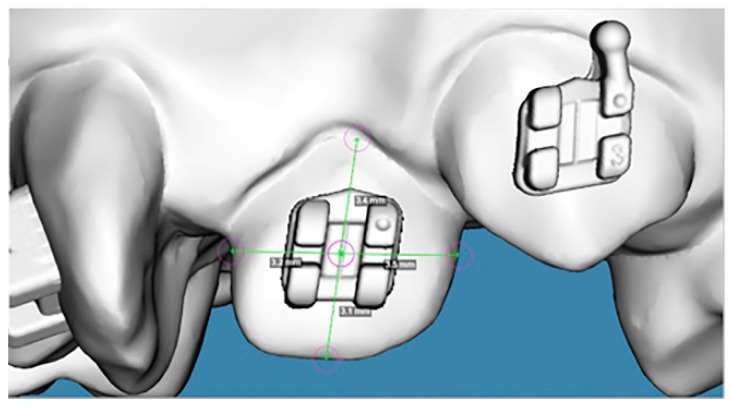
Four parameters were measured from the midpoint of each bracket to the: mesial margin (CM: center mesial), distal margin (CD: center distal), occlusal/incisal margin (CI: center incisal/occlusal), and gingival margin (CG = center gingival). The horizontal line was parallel to the brackets’ wing and perpendicular to its long axis.

Intraoral scans were taken after IDB using an iTero Element scanner (Align Technology, San Jose, CA, USA) (Figure 4). The scanned IDB models were transferred to Dolphin Imaging software V 11.95 (Dolphin Imaging and Management Solutions, Chatsworth, CA, USA) to perform the selected measurements. Four points were selected for each tooth: from the midpoint of each bracket to the mesial margin (CM: center mesial), distal margin (CD: center distal), occlusal/incisal margin (CI: center incisal/occlusal), and gingival margin (CG = center gingival) ensuring that the line formed between the two points was parallel to the brackets’ wing and perpendicular to its long axis (Figure 5).

The sample size needed for conducting this study was calculated using the G*Power 3.1 software. A sample size of 80 achieved 95% power with α = 0.05 and effect size 1.76.

Reliability was determined by repeating the set of measurements twice for 200 brackets where the second round of measurements was performed by the same investigator after one week. Intraclass correlation coefficients (ICCs) along with 95% confidence intervals were calculated. Data were entered into the computer using IBM-SPSS for Windows version 28.0 (SPSS Inc., Chicago, IL, USA). The Shapiro–Wilk test was used to test the normality of continuous variables. Mann–Whitney test was used to compare the means between two groups. When comparing the means between more than two groups the Kruskal–Wallis test was used. A *p*-value of less than 0.05 was considered significant.

## 3. Results

Intra-examiner reliability was high within each group as assessed by ICC (ICC ≥ 0.90 for all measurements). In group 1, statistically significant differences were detected between the one-piece, segmented, and control groups for CD12, CD21, and CI25 (*p* < 0.001), while the remaining measurements showed no significant differences (*p* = 1.0) (Table 1). Non-significant differences were detected for CI15, CI12 (*p =* 0.130), CM14, CG12, CI11, CI21, CM21 (*p* = 0.374) and CD24, 0.229 for CI22, and 0.384 for CM22 (*p* = 0.394) as shown in Table 1. In group 2, no significant differences were detected between the one-piece, segmented and control (*p* > 0.05) for all measurements (Table 2).

## 4. Discussion

The ideal bracket positioning minimizes the time and expense of repositioning brackets midway through treatment [16,17]. Since similar bracket prescriptions can translate differently depending on its bonding position on the tooth, it is crucial that brackets are placed correctly in all dimensions. A positioning error of a bracket in one dimension can change the tooth’s torque and buccolingual position [18]. Because brackets work simultaneously through the wire, one poorly positioned bracket can affect adjacent brackets, and the effect is multiplied when more than one bracket is misplaced, preventing the case from being efficiently finished [19].

The advancement of IDB technology within the last decade has offered benefits for clinicians by reducing chair time and improving bracket placement accuracy. Compared to direct bonding, IDB was reproducibly more accurate for bracket positioning with fewer torque and rotation errors [20,21]. The IDB also reduced chances for plaque buildup around the brackets and the decalcified white spots [22]. However, this technology has several drawbacks, such as the use of expensive materials and labor-intensive laboratory processes [23].

IDB utilizing digital technology provides the benefits of traditional IDB, as well as featuring faster processing, computer-assisted model analysis, more accurate bracket placement, standardization, ease of fabrication, and fewer manufacturing steps [24,25]. The present study compared the accuracy of IDB techniques in moderate and severe crowding cases using 3D printed one-piece and segmented transfer trays. The results indicated non-significant differences in the transfer accuracy of the IDB using one-piece and segmented transfer trays in moderate and severe malocclusion, except in moderate crowding, significant differences were detected at CD12, CD21, and CI25.

Kim et al. compared the digital bracket positions after IDB of five dental models with intended digital bracket positions using a 3D digital program. Their results showed high transfer accuracy in the linear dimensions (93–100% within acceptable range), and low transfer accuracy in the angular dimensions (43–73% within acceptable range) using 3D-printed jigs in vitro [26]. Chaudhary et al. reported low magnitudes and rates of in vivo transfer errors in the linear dimensions when using 3D-printed transfer trays [27]. The high transfer accuracy in the linear dimensions may be due to the relatively rigid nature of the printed tray material [13]. Later studies have attributed the weak angular control of bracket positioning to inconsistencies in the amount of adhesive used and angular bonding failure caused by the tray design for hook and undercut relief [28,29]. This suggests that thicker and more rigid transfer trays can improve angular control. However, this may result in patient discomfort and higher bond failure rates due to difficulties in removing the trays after curing. In the current study, we used a modified design of transfer trays that were rigid enough to hold the brackets in the predetermined position by only covering only the buccal aspect of the teeth.

In this study, before bonding, adhesive material was manually applied to the bracket base. Inconsistencies in bracket positioning may arise from the non-standardized amount of adhesive used which was subject to the discretion of the operator. A way to overcome this factor is by using pre-coated brackets. To avoid separation from the build platform during printing, different support structures are necessary for different angulations. Arnold et al. [30] discovered that the arrangement of objects on the build platform of SLA printers influences accuracy. They found that the best accurate models are created in the front of the platform. Unlovskiy et al. [31] on the other hand, discovered that objects placed in the center of the build platform are more accurate than those placed on the edge. In our study, the transfer trays covered the whole build platform according to their size and available space.

Bracket debonding during IDB has been previously reported due to the possibility of bracket position, weak curing technique, and/or improper design/materials of bracket transfer trays [32]. Bond failures in this study were almost exclusively related to bracket embedment in the tray material. A total of 6 deboned brackets recorded were excluded from the study. They were identified as two from each group of one-piece, and one from each group of segmented trays, providing a ratio of 2:1 (one-piece to segmented). This was mainly due to the difficulty in removing the one-piece tray. It is possible that trimming and reducing tray thickness could lower bond failure rates but might result in less accuracy due to less rigidity. The transfer tray should be flexible enough not to cause any damage or bonding failure to the brackets during removal. In our study, the resin material used in 3D printing was semi-rigid with 0.05 mm thickness, successfully securing the brackets’ position during bonding with some flexibility to avoid damaging bonded brackets during tray removal. Although the number of deboned brackets was greater for the one-piece tray than for the segmented tray, no statistically significant differences were found in the number of deboned brackets among the groups.

While identifying landmarks for measuring bracket position accuracy on scanned models, the borders of the bracket base were not clear, possibly impacting the linear dimension. Instead, the midpoint of the bracket base was considered in this study as the reference landmark. Distances were measured from the tooth margins (CI, CG, CM, and CD) to the midpoint of each bracket. In addition, during scanning with the iTero scanner, which operates on the theory of light emission, the metallic brackets scatter light rays, causing apparent multiplanar surfaces on the digital post-bonding scans. A review of the commercially available intraoral scanners revealed that the iTero scanner, used in this study, has the best precision [33].

The shape of the bracket may not be accurately reproduced by intraoral scanners as the scanned images represent some distortion around the brackets. The distortion may take place because of the reflective surfaces of the brackets. However, the in vivo situation is much different because of the moisture and the accessibility to the posterior tooth surface [34]. A study by Kang et al. confirmed the accuracy of intraoral scans in the presence of brackets and recommended that regions beyond 0.50 mm around the brackets can be used for superimposition on images without brackets. They concluded that the amount of distortion is clinically acceptable [34]. This supports the idea that the distortion produced by the intraoral scanners may negatively affect the angular measurements, but the linear measurements are not affected.

Bracket position could be influenced by the variability in tray seating, adaptation, ease of removal and the support of the 3D-printed tray. In this study, prior to bonding, the adhesive material was manually placed on the bracket base. Errors in bracket positioning could be a result of the non-standardized amount of adhesive material that was left to the operator’s discretion. We consider this a limitation in the current study. This can be overcome by using pre-coated brackets. Another limitation was not using the molar teeth for comparisons. This study did not include the molars in the IDB. Compared to anterior brackets, we have found that posterior brackets are more prone to bonding failure. Finally, the clinical situation in IDB intraorally is likely to be quite different from the laboratory scenario, particularly regarding difficulty seating the tray in crowded arches.

The findings of the present study can directly influence clinical protocols by identifying the most effective tray design, leading to better bracket placement accuracy. This improvement can enhance treatment outcomes and reduce the need for adjustments, making orthodontic treatments more efficient. Lack of clinical trials is a limitation. Future planned studies should focus on validating the reliability of IDB using the one-piece and segmented transfer trays in vivo and analyzing the effects of additional factors that could present clinical challenges such as restricted mouth opening and muscle movement, patient compliance, moisture control, and soft tissue interference.

## 5. Conclusions

One-piece and segmented 3D printed transfer trays are considered accurate tools for IDB in moderate and severe malocclusion cases. The severity of crowding did not affect the accuracy of bracket transfer in indirect bonding.

## Figures and Tables

**Table 1:** Comparison of the accuracy of bracket positions among the groups in moderate crowding (Group 1).

Parameter	Control	Full	Segmented	*p*-Value
	Mean (SD)	Mean (SD)	Mean (SD)	
CG15	2.6 (0.0)	2.6 (0.0)	2.6 (0.0)	1.00
CI15	3.7 (0.03)	3.7 (0.0)	3.7 (0.0)	0.13
CM15	2.6 (0.0)	2.6 (0.0)	2.6 (0.0)	1.00
CD15	3.8 (0.0)	3.8 (0.0)	3.8 (0.0)	1.00
CG14	3.3 (0.0)	3.3 (0.0)	3.3 (0.0)	1.00
CI14	4.1 (0.02)	4.1 (0.0)	4.1 (0.0)	1.00
CM14	3.3 (0.0)	3.3 (0.0)	3.3 (0.0)	0.37
CD14	3.7 (0.0)	3.7 (0.0)	3.7 (0.0)	1.00
CG13	3.7 (0.0)	3.7 (0.0)	3.7 (0.0)	1.00
CI13	5.1 (0.0)	5.2 (0.0)	5.0 (0.0)	1.00
CM13	3.2 (0.0)	3.2 (0.0)	3.2 (0.0)	1.00
CD13	5.1 (0.0)	5.1 (0.0)	5.1 (0.0)	1.00
CG12	6.1 (0.02)	6.1 (0.0)	6.1 (0.0)	1.00
CI12	4.2 (0.03)	4.1 (0.0)	4.0 (0.0)	0.13
CM12	3.2 (0.0)	3.2 (0.0)	3.2 (0.0)	1.00
CD12	3.5 (0.0)	3.4 (0.0)	3.4 (0.0)	<0.001 *
CG11	5.4 (0.0)	5.4 (0.0)	5.4 (0.0)	1.00
CI11	4.5 (0.02)	4.5 (0.0)	4.5 (0.0)	0.37
CM11	4.2 (0.0)	4.2 (0.02)	4.2 (0.0)	1.00
CD11	3.7 (0.0)	3.7 (0.0)	3.7 (0.0)	1.00
CG21	6.5 (0.0)	6.5 (0.0)	6.5 (0.0)	1.00
CI21	4.6 (0.02)	4.6 (0.0)	4.6 (0.0)	0.37
CM21	4.4 (0.0)	4.4 (0.02)	4.4 (0.0)	0.37
CD21	4.1 (0.0)	4.1 (0.0)	4.1 (0.0)	<0.001 *
CG22	5.4 (0.0)	5.4 (0.0)	5.4 (0.0)	1.00
CI22	4.4 (0.02)	4.4 (0.02)	4.4 (0.0)	0.23
CM22	3.3 (0.02)	3.3 (0.02)	3.3 (0.0)	0.38
CD22	3.6 (0.02)	3.6 (0.0)	3.6 (0.0)	1.00
CG23	3.1 (0.0)	3.1 (0.0)	3.1 (0.0)	1.00
CI23	5.3 (0.0)	5.3 (0.0)	5.3 (0.0)	1.00
CM23	3.6 (0.0)	3.6 (0.0)	3.6 (0.0)	1.00
CD23	4.3 (0.0)	4.3 (0.0)	4.3 (0.0)	1.00
CG24	3.3 (0.0)	3.3 (0.0)	3.3 (0.0)	1.00
CI24	3.8 (0.0)	3.8 (0.0)	3.8 (0.0)	1.00
CM24	3.7 (0.0)	3.7 (0.0)	3.7 (0.0)	1.00
CD24	4.0 (0.02)	4.1 (0.0)	4.0 (0.0)	0.39
CG25	2.5 (0.0)	2.7 (0.0)	2.5 (0.0)	0.10
CI25	3.2 (0.02)	3.3 (0.0)	3.3 (0.0)	<0.001 *
CM25	3.0 (0.00)	3.0 (0.0)	3.0 (0.0)	0.10
CD25	3.0 (0.00)	3.0 (0.0)	3.0 (0.0)	0.10

* Significant at *p* ≤ 0.001.

**Table 2:** Comparison of the accuracy of bracket positions among the groups in severe crowding (Group 2).

Parameter	Control	Full	Segmented	*p*-Value
	Mean (SD)	Mean (SD)	Mean (SD)	
CM15	4.0 (0.0)	4.0 (0.0)	4.0 (0.0)	0.10
CD15	2.5 (0.0)	2.5 (0.0)	2.5 (0.0)	1.00
CG14	2.6 (0.0)	2.6 (0.0)	2.6 (0.0)	0.10
CI14	3.9 (0.0)	3.9 (0.0)	3.9 (0.0)	1.00
CM14	4.0 (0.0)	4.0 (0.0)	4.0 (0.0)	1.00
CD14	3.7 (0.02)	3.7 (0.0)	3.7 (0.0)	0.37
CG13	3.5 (0.0)	3.5 (0.0)	3.5 (0.0)	1.00
CI13	4.5 (0.0)	4.5 (0.0)	4.5 (0.0)	0.10
CM13	3.6 (0.1)	3.6 (0.0)	3.6 (0.0)	0.13
CD13	4.1 (0.0)	4.1 (0.0)	4.1 (0.0)	1.00
CG12	3.4 (0.0)	3.4 (0.0)	3.4 (0.0)	1.00
CI12	3.4 (0.0)	3.4 (0.0)	3.4 (0.0)	1.00
CM12	3.5 (0.0)	3.5 (0.0)	3.5 (0.0)	1.00
CD12	3.5 (0.0)	3.5 (0.0)	3.5 (0.0)	1.00
CG11	4.6 (0.0)	4.6 (0.0)	4.6 (0.0)	1.00
CI11	4.4 (0.0)	4.4 (0.0)	4.4 (0.0)	1.00
CM11	4.5 (0.0)	4.5 (0.0)	4.5 (0.0)	1.00
CD11	4.5 (0.0)	4.5 (0.0)	4.5 (0.0)	1.00
CG21	3.4 (0.03)	3.4 (0.0)	3.4 (0.0)	0.37
CI21	4.7 (0.0)	4.7 (0.0)	4.7 (0.0)	1.00
CM21	4.2 (0.0)	4.2 (0.0)	4.2 (0.0)	1.00
CD21	4.5 (0.0)	4.5 (0.0)	4.5 (0.0)	1.00
CG22	3.4 (0.02)	3.4 (0.0)	3.4 (0.0)	0.37
CI22	3.1 (0.0)	3.1 (0.0)	3.1 (0.0)	1.00
CM22	3.2 (0.0)	3.2 (0.0)	3.2 (0.0)	1.00
CD22	3.5 (0.0)	3.5 (0.0)	3.5 (0.0)	1.00
CG23	3.0 (0.02)	3.0 (0.0)	3.0 (0.0)	0.37
CI23	3.4 (0.3)	3.8 (0.0)	3.8 (0.0)	0.15
CM23	3.4 (0.0)	3.4 (0.0)	3.4 (0.0)	1.00
CD23	3.9 (0.0)	3.9 (0.0)	3.9 (0.0)	1.00
CG24	2.5 (0.0)	2.5 (0.0)	2.5 (0.0)	1.00
CI24	3.4 (0.0)	3.4 (0.0)	3.4 (0.0)	1.00
CM24	3.7 (0.0)	3.7 (0.0)	3.7 (0.0)	1.00
CD24	4.0 (0.02)	4.0 (0.0)	4.0 (0.0)	0.37
CG25	2.2 (0.0)	2.2 (0.0)	2.2 (0.0)	1.00
CI25	3.5 (0.02)	3.5 (0.0)	3.5 (0.0)	0.39
CM25	3.5 (0.0)	3.5 (0.0)	3.5 (0.0)	1.00
CD25	3.3 (0.0)	3.3 (0.0)	3.3 (0.0)	1.00

## Data Availability

The datasets generated and analyzed during the current study are available from the corresponding author on reasonable request.
